# Specialised minds: extending adaptive explanations of personality to the evolution of psychopathology

**DOI:** 10.1017/ehs.2022.23

**Published:** 2022-05-31

**Authors:** Adam D. Hunt, Adrian V. Jaeggi

**Affiliations:** Institute of Evolutionary Medicine, University of Zürich, Switzerland

**Keywords:** Evolutionary psychiatry, neurodiversity, evolutionary psychology, hunter-gatherers, personality

## Abstract

Traditional evolutionary theory invoked natural and sexual selection to explain species- and sex-typical traits. However, some heritable inter-individual variability in behaviour and psychology – personality – is probably adaptive. Here we extend this insight to common psychopathological traits. Reviewing key findings from three background areas of importance – theoretical models, non-human personality and evolved human social dynamics – we propose that a combination of social niche specialisation, negative frequency-dependency, balancing selection and adaptive developmental plasticity should explain adaptation for individual differences in psychology – ‘specialised minds’ – explaining some variance in personality and psychopathology trait dimensions, which share various characteristics. We suggest that anthropological research of behavioural differences should be extended past broad demographic factors (age and sex) to include individual specialisations. As a first step towards grounding psychopathology in ancestral social structure, we propose a minimum plausible prevalence, given likely ancestral group sizes, for negatively frequency-dependent phenotypes to be maintained as specialised tails of adaptive distributions – below the calculated prevalence, specialisation is highly unlikely. For instance, chronic highly debilitating forms of autism or schizophrenia are too rare for such explanations, whereas attention-deficit-hyperactivity disorder and broad autism phenotypes are common enough to have existed in most hunter-gatherer bands, making adaptive explanations more plausible.

## Graphical abstract

**Figure:**
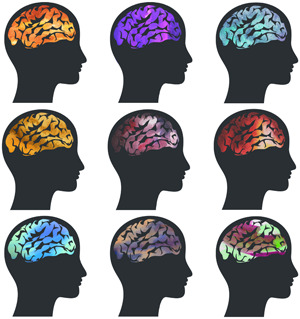

**Social media summary:** Traits of personality and psychopathology could result from a shared evolutionary process of cognitive specialisation.

## 1. Introduction

Under traditional evolutionary theory, heritable phenotypic variation is expected to be positively selected until fixation or negatively selected until elimination (Buss & Hawley, 2011: ix). Following this, evolutionary psychology has primarily accounted for psychological mechanisms in terms of evolved universal cognitive architecture (Lukaszewski et al., 2020), with adaptive individual variation arising owing to plastic reactions to individual circumstances. This approach is visible in Darwin's *The Expression of the Emotions in Man and Animals*, framing emotional and behavioural responses as shared adaptive strategies. Personality, defined in behavioural ecology as repeatable individual differences in behaviours (Dochtermann et al., 2015) and defined more broadly in psychology as those characteristics of individuals that describe and account for consistent patterns in feeling, thinking and behaving (Gosling, 2001: 46), has thus often been portrayed as noise – neutral or maladaptive variation around an optimum (Tooby & Cosmides, 1990). This perspective is now changing, as growing theoretical and empirical research in humans and non-humans finds evidence for adaptive individual differences.

Human personality is moderately heritable; stable over time, context and culture; shows continuities with non-human personality differences; provides predictive power in forecasting behaviour; and has consequences on evolutionarily relevant factors such as survival, mating success, status, fecundity and parenting (Buss & Hawley, 2011: x). As heritable phenotypes active during reproductive years are visible to selection, an evolutionary explanation is required. Psychologists and psychiatrists also seek evolution-informed explanations for mental disorders (Del Giudice, 2018; Nesse, 2019). Personality and psychopathology overlap substantially – personality dimensions are related to specific disorders (Widiger et al., 2017), and many mental disorders are relatively common, heritable and affect fitness, requiring an evolutionary explanation (Keller & Miller, 2006). Just as personality differences are reflected by gradual neurobiological differences, common mental disorders do not generally have discrete pathological causes, and descriptivist approaches categorising mental disorders into distinct diagnoses ignore the reality that mental disorders are dimensional, sharing symptoms and alleles with the subclinical population (Geschwind & Flint, 2015; Zachar & Kendler, 2017). Even the term ‘psychopathology’ is misleading in implying identified disease – to follow past literature this paper uses it, but we support more cautious use of medicalising language in psychiatry, as seen in ‘neurodiversity’ perspectives (Kapp et al., 2020), which better align with the evidence and theory presented here.

This paper is concerned with evolutionary explanations for relatively *stable* individual differences in personality and psychopathology: ‘traits’ rather than ‘states’. The question is why individuals differ in these traits, and why such differences could lead to diagnosable psychopathology. This excludes common emotions or mood disorders of anxiety and depression, although individual differences in traits often manifest in differential propensity towards certain states (e.g. neuroticism is related to commonly experiencing anxiety; psychopathy as experiencing less empathy, guilt or shame). Throughout this paper we shall examine evidence and theory that indicate that traits of personality and psychopathology are disciplinarily separated but biologically related phenomena, in need of related evolutionary explanation. Although wary of the mistake of overfitting multiple phenotypes into a single model, we believe that the idea that human evolution has selected for *specialised minds* (Tooby & Cosmides, 1996; Troisi, 2005) is most useful – like Dall et al. (2012), we believe that the concept of specialisation nicely encompasses the multiple processes leading to adaptive individual differences in behaviour. We shall examine evidence indicating that specialised minds should have adapted around social niche sizes and dynamics, and be sensitive to local ecology and culture (Smaldino et al., 2019). Social niche specialisation, social selection, adaptive developmental plasticity, negative frequency-dependency and temporally fluctuating selection should lead to complex specialising fixed and plastic effects manifesting via innate predispositions and developmental responses (Section 2). Specialisations involve trade-offs and functional and dysfunctional manifestations or aspects, often context-dependent, with stabilising costs and benefits, potentially maintained via inclusive fitness (Sections 4 and 5). Heterogeneity is the rule rather than the exception; recognition of pathological, neutral and adaptive forces will be required in full evolutionary explanations, especially of psychopathology (Section 5). Our main argument shall be that specialisation is the most useful adaptive process for theorists to consider, and that taking into account theoretical models (Section 2), non-human personality (Section 2), human evolved psychology and social dynamics (Section 3), and evolutionary approaches to personality (Section 4) will prove crucial in explaining psychopathological traits (Section 5). We finish by suggesting productive avenues for future research, especially by examining anthropological research with the proposed theoretical models in mind (Sections 6–8).

## 2. Evolutionary models of individual differences and examples in non-human animals

Adaptive individual differences in phenotype can arise via various mechanisms and selection pressures. Plasticity can adaptively match phenotypes to different environments (West-Eberhard, 2003), whilst maintenance of genetically based alternative strategies requires equal fitness over multiple generations (Smith, 1976). After briefly summarising relevant fixed genetic and plastic effects, we shall consider selective forces for phenotypic specialisations.

### Genetic effects on individual differences

At the genetic level, individual differences have four possible explanations (Keller et al., 2011):

1. *Neutrality.* Genetic drift is maintaining differences; they have a negligible effect on reproductive success and are under neither positive nor negative selection.
2. *Mutation-selection balance.* Individual differences are caused by harmful mutations which arise too frequently to be completely removed by negative selection.
3. *Positive selection on recent mutations.* Responsible variants are under positive selection but have arisen too recently to have reached fixation.
4. *Balancing selection.* The variant's fitness depends on conditions, with none being optimal for long enough to reach fixation (at the phenotypic level the process may be negatively frequency-dependent, or temporally or spatially dependent, or sexually antagonistic).

Of these, only under balancing selection is individual genetic variation itself selected to produce alternative strategies.

### Plasticity and environmental effects on individual differences

Behavioural ecology starts from the assumption that behaviour is optimal in a given situation (Nettle et al., 2013). The presence of individual differences in behaviour which ‘hang together’ in stable personality types (also called ‘behavioural syndromes’) is therefore a fundamental problem (Dall et al., 2004; Sih et al., 2004). Why would some individuals be more aggressive than others, and why would an aggressive individual show differences in their interactions with conspecifics *and* foraging behaviour *and* exploratory behaviour, rather than acting facultatively?

One reason evolution can favour behavioural consistency is because social interactions become predictable, allowing social partners to learn and adaptively respond to personality types (Wolf et al., 2011). This especially occurs when individuals coordinate actions or have a mutual interest to avoid certain outcomes. Variation in turn favours responsiveness to consistent personalities, so a coevolutionary process can occur between responsiveness and consistency (McNamara et al., 2008). The ability to learn a skill and occupy a particular niche (see below) also encourages plasticity canalising over time. Potential costs of plasticity exist (Ellis & Del Giudice, 2019); cues received from the environment take time to sample and react to, may be misleading and difficult to reverse, and plasticity itself may be energetically costly. Adaptive plasticity is not always an optimal or possible strategy.

Relevantly to psychopathology, adaptive developmental plasticity and conditional adaptation leading to individual differences in phenotype have been studied and theorised about extensively in relation to stress (Ellis & Del Giudice, 2019). Assumptions that high-stress environments simply dysregulate biology to cause dysfunction are misplaced – adaptive reactions to stressful environments to maximise expected fitness are expected (e.g. Thayer *et al.*, 2018), sometimes involving trade-offs in organism longevity or health, as in certain applications of life history theory (see Section 5).

### Key selective forces encouraging individual differences in behaviour

Individual differences in behaviour often occur in social contexts (Dall et al., 2012). ‘Social selection’ is a relevant process of natural selection involving the fitness effects of social behaviours and social competition (and subsumes sexual selection; Lyon & Montgomerie, 2012; Nesse, 2007; West-Eberhard, 1979). Organisms affect conspecifics, and the potential fitness benefits of relationships and group membership cause social selection for traits optimising access to these resources. This can lead to cooperation with unrelated individuals, apparent altruism and systems of reward and punishment (Barclay, 2013). Importantly, developing valuable specialised skills and attributes and being individually recognised for them is a key way for humans to ingratiate themselves with cooperators (Tooby & Cosmides, 1996; see Section 3).

Social niche specialisation involves individuals in a population occupying different roles (Bergmüller & Taborsky, 2010; Montiglio et al., 2013) and is a key explanation for individual differences in social species. Non-human experimental evidence indicates that plastic social niche specialisation leads to various lasting behavioural changes: house mouse sibling sex ratio affects behavioural flexibility, great tit food rationing of siblings affects exploration behaviour, and rearing cichlids with or without dominants affects aggressive and submissive behaviours (Bergmüller & Taborsky, 2010). Genetic and environmental effects can interact in social niche specialisation; for example, an initial genetic predisposition and early life circumstances can lead to an individual occupying a certain niche, at which point they maximise fitness by plastically developing to maximise their efficacy in that niche (Smaldino et al., 2019). Multiple factors can affect niche selection, including state dependence (the individual's states predispose towards success in certain niches), frequency-dependence (negative if niches have limited resources or positive if niches’ resources are maximised by multiple occupancy) and social awareness and eavesdropping (where conspecifics are aware of an individual's niche and this makes continually occupying the niche more profitable).

Intraspecific trait variation affects the ecological dynamics of communities in multiple relevant ways (Bolnick et al., 2011). Increasing environmental heterogeneity and population density increase personality diversity in small mammals including deer mice, southern red-backed voles and northern short-tailed shrews (Mortelliti & Brehm, 2020). An increased degree of variation makes a population more generalised via individual specialisation, reducing competition between members and increasing species niche width (e.g. in diet; Bolnick et al., 2003) as the community extracts more resources from the environment. Such species are more robust to extinctions. Social structures in humans can integrate personality differences into a ‘pool of competence’ (Wolf & Krause, 2014), as collectives of differentiated individuals increasing individual and group fitness through cooperation. Some researchers even suggest that the strength of this cooperative effect makes other evolutionary pathways of explaining diversity unnecessary (Nonacs & Kapheim, 2007) and that computational challenges of specialising played a significant role in selection pressures for *Homo* cognitive abilities (Hagen et al., 2021). The division of behaviour then encourages ongoing cooperation (Bergmüller et al., 2010; McNamara & Leimar, 2010). Paradigmatic examples are in eusocial insects, where division of labour and specialisation amongst workers increase colony efficiency, and emerges spontaneously in small groups of morphologically similar workers (Ulrich et al., 2018).

The balancing of selection effects on psychological traits can arise from temporally fluctuating selection (Taylor et al., 2014) when exogenous environmental (e.g. food availability, disease, conflict) and endogenous population factors (e.g. sex ratios; Del Giudice, 2012) change, affecting optimal behaviour, but never bringing a single phenotype or genotype to fixation. Temporally fluctuating selection has historically been questioned for its sufficiency in maintaining variation (but see Del Giudice, 2020: S2), with negative frequency-dependent selection the preferred explanatory force explaining adaptive individual variation via balancing selection (potentially mistakenly ignoring alternative or co-occurring forces; Brisson, 2018). Frequency-dependent selection occurs when the fitness of a genotype or phenotype depends on its frequency in the population. In positive frequency-dependency, higher frequency results in higher fitness. Under negative frequency-dependency, higher frequency results in lower fitness; this can lead to individual differences stabilising in frequency. Negative frequency-dependency is considered a key process maintaining individual variation in populations, exemplified in alternative mating strategies such as ‘sneaky’ males which imitate females (in isopods, sunfish, garter snakes and shorebirds) or providing exceptional care to young (cichlid fish; Shuster, 2010). Behavioural strategies include attempting to woo females with gifts or alternatively attempting to mate by force, or varying in tendency to seek multiple partners or defend a single partner. These alternative strategies may be plastic or inherited and fixed. Although negative frequency-dependency is often modelled in a simplified manner with few, discrete morphs (e.g. hawk and dove; male and female), it can also maintain multiple morphs on a continuous distribution underpinned by multiple polygenic factors, allowing species to fill niches as they become available (Slatkin, 1979). Note that forces of balancing selection are not mutually exclusive; negative frequency-dependency and temporally fluctuating selection can act simultaneously in maintaining variation. Combining the effects of these forces is realistic – individual differences’ adaptiveness depending both on fluctuating environmental factors and group composition where rare traits are advantageous.

Theoretical models of adaptation account for regular maladaptive outcomes, although maladaptation or dysfunction is only ‘disorder’ if deemed harmful by social consensus (Wakefield, 2015). Evolutionary medicine and psychiatry have identified various ultimate causes of disorders (Nesse, 2005, 2019). These may be broadly classified as genuine dysfunctions; functional mechanisms mismatched to modern environments and currently maladaptive; or generally adaptive traits with maladaptive or undesirable consequences (Del Giudice, 2018, p133). Medically relevant harm can have adaptive causes, and as noted by Troisi (2005), selection for alternative strategies could be a fundamental evolutionary cause of psychopathology. Adaptive dimensional strategies can lead to maladaptive extremes; costs of developmental plasticity could be maladaptive manifestation in a certain proportion of cases; assortative mating between similar individuals may lead to overexpression of an ancestrally adaptive trait; and mismatch may cause adaptations to manifest maladaptively in certain environments, or be defined as disorders by modern psychiatry. Heterogeneity both between and within disorder categories means that they require explanation via multiple models of adaptation and processes of maladaptation or dysfunction (Del Giudice, 2018).

### Implications of theoretical and non-human literature in explaining human individual differences

The theoretical and non-human literature unequivocally shows that assumptions that all individual differences in behavioural traits are noise awaiting extinction or fixation are no longer tenable. However, complex dynamics maintain individual differences theoretically and in non-human species (e.g. Bergmüller and Taborsky, 2010; Shuster, 2010; Mouchet et al., 2021). Theoretical models explain different *aspects* of biological phenomena, so specific models may be simultaneously (but probably not equally) explanatory – models are not only potentially compatible but *necessarily* co-occur. Negative frequency-dependency often has effects in social niche specialisation (but see Brisson, 2018). Models of genetic and environmental effects are required simultaneously. Models of adaptive dimensional traits include functional and dysfunctional manifestations. The impossibility of full explanations with single models is a point against overreliance on single frameworks such as life history theory, which has been suggested as a unifying framework for evolutionary psychopathology (Del Giudice, 2018), but is under scrutiny for its somewhat idiosyncratic applications to humans (Frankenhuis & Nettle, 2020). Explaining individual differences in human psychological traits, adaptive and maladaptive, requires integrating a multiplicity of models.

## 3. Evolved human psychology and social dynamics

Theoretical models of evolved individual differences overwhelmingly depend on social dynamics. Here we consider various lines of evidence pointing towards the likelihood of humans evolving adaptive, relatively stable inter-individual differences in feeling, thinking and behaving. After noting aspects of human psychology which particularly encourage inter-individual specialisation, we consider anthropological evidence on hunter-gatherer skill niches and social structure, and group sizes and interaction dynamics (to later consider the implications of manifestation in ancestral groups; Section 7).

As reviewed above, specialisations are observed in non-human species and predicted by theoretical models under certain circumstances. Those circumstances are not just present, but heightened in human psychology. Human cognition shows high plasticity, allowing specialisation and canalisation which can't be easily reverted or copied; a tendency towards developing individual skills (Tooby & Cosmides, 1996); and behavioural flexibility and intelligence to select oneself into one's optimum available niche and out of inappropriate niches (Hooper et al., 2015). Humans seem to have evolved excellent capacities for noticing and acting upon other people's individual differences (Buss, 1996; Buss & Hawley, 2011), allowing intelligent partner selection, cheater detection and knowledge of others’ social niches. Forces of social selection and negative frequency-dependent selection may be heightened by human cognitive ability to assess, remember, compare and share information about individuals (Penke, 2010), allowing social status to be ascribed to individuals who optimise group functioning, encouraging frequency-dependent division of labour and cognitive specialisation, widening the human ecological niche. These capacities provide perhaps unparalleled social selection for specialisation in humans. The fact that modern economies are run with division of labour and specialisation as a first principle, and that most modern specialisation is cognitive, with different personality profiles suiting different careers (Denissen et al., 2018; Wilmot & Ones, 2019), may be a formalised continuation of a long evolutionary history of social niche specialisation which contributed to human ecological success.

### Human ancestral social organisation: dynamics, group sizes and niches

Accounting for the evolution of human specialised minds needs reference to ancestral social organisation, and thus evidence from anthropology, particularly of hunter-gatherer societies. Whilst the antiquity of this social organisation cannot be known with certainty, at least some elements of it arguably extend back some 2 million years to *Homo ergaster* (Willems & van Schaik, 2017).

Nomadic hunter-gatherer societies are generally egalitarian, sharing food and cooperating on daily tasks; however, social status does differ between individuals and has many fitness-relevant benefits (Von Rueden, 2014; Von Rueden et al., 2008; Von Rueden & Jaeggi, 2016), making status allocation a force of social selection. Reputations for competency and pro-sociality earn social support and attract cooperative partners, particularly in the contexts of collective labour (Macfarlan & Lyle, 2015). Indeed, cooperation earns social status to such an extent that social status allocation has been proposed as a key factor encouraging the evolution of human cooperative behaviour (Von Rueden et al., 2019).

Traits selected as social-niche specialisations and under negative frequency-dependency evolve in the context of niche size, species-typical group size and interaction dynamics with conspecifics. Hunter-gatherer group structure is typically composed of residential units (‘bands’), interacting, migrating and exchanging spouses with other bands in metagroups (‘tribes’). Individuals can choose to join foraging groups to optimise their relative efficiency (Smith, 1985) and assort into bands to optimise co-ordination and complementarity whilst maximising personal productivity (Hooper et al., 2015). Typical bands have a mean size of 28 individuals (Hill et al., 2011), consisting of about half a dozen adult male and female couples, children of various ages and perhaps a few post-reproductive individuals. Despite large variation in ecology and other aspects of behaviour, hunter-gatherer band sizes are remarkably similar (Kelly, 2013), probably reflecting consistent constraints such as the need to buffer shortfalls through food sharing (Winterhalder, 1986). Tribe size is more variable than band size, and bands may interact in complex networks of multiple languages that defy the notion of small-scale society (Bird et al., 2019). A relevant group size for our purpose is seen in periodic aggregations of bands who exchange marriage partners (to avoid inbreeding) and material goods, with a geometric mean of 165 individuals (95% confidence limit, 152–181; Hamilton et al., 2007; although the range extends from tens to several hundred individuals). These larger pools of social partners affects gene flow and the flow of information – foragers can pass information to and learn from potentially hundreds of individuals in a lifetime (Hill et al., 2014; Salali et al., 2016).

Traditional human societies provided a breadth of social niche possibilities, empowered by individual ability to migrate or settle into optimally suitable groups given their circumstances (Hooper et al., 2015). Ancestral social niches could have been explicit (e.g. shamanism) or implicit (e.g. personality types which ‘fit in’ with conspecifics). Explicit niches are most recognisable in division of labour and noticeable individual skills and talents (Sugiyama & Sugiyama, 2003) as avenues to gaining social status and cooperating (Jaeggi et al., 2016; Macfarlan & Lyle, 2015). Hunter-gatherer division of labour shows patterns between gender and age (Bird & Codding, 2015; Gurven & Hill, 2009). Most stereotypically, males more regularly hunt large prey, whilst females gather plant foods and engage in childcare. However, this common demographic patterning hides potentially relevant diversity – for example, the Aché note the roles of finder, caller and killer whilst cooperatively hunting (Walker et al., 2002), which may suit different cognitive abilities, despite occurring within a gender-specific activity.

Sugiyama and Sugiyama (2003) extensively list possible niches for hunter-gatherer individuals to occupy. They may be in social roles (e.g. shaman or chief), specific crafts (e.g. pottery, basket weaving, boat building), or other performance or art (storytelling, singing, composition, dance). Social roles go beyond particular skills, for example in cooperators who aid in warfare, punishment or generosity. Knowledgeable individuals in areas of geography, spirituality, medicine and subsistence and technological skills are acknowledged (Lightner et al., 2021b). Individual roles are recognised and often admired: differential quality of manufactured tools is noted (Sugiyama & Sugiyama, 2003), as is efficacy in storytelling or teaching (Smith et al., 2017). Different abilities between specialists are tracked, and the best individuals are acknowledged, earning valuable social support (Schniter et al., 2018; Smith et al., 2017). Individual skills and personality traits are always judged in comparison with the immediate group; exceptionality exists in comparison with others (Tooby & Cosmides, 1996) and the best specialist in a niche can be disproportionately valued. For example, Singh and Henrich (2020) found that across two villages a single shaman performed 32% of ceremonies, the next best performed 14% and the remaining 54% were spread (unevenly) across 37 other individuals.

Different niches (e.g. tool use, shamanism and oratory skill) probably involve different dynamics, and the biological feasibility for optimal specialisation may differ between niches (e.g. shamanistic niche filling may optimise via adaptive developmental plasticity decided by early life experience, whilst tool-making niches may optimise via heritable fixed components). Precise dynamics are undoubtedly complex. For example, social niches in shamanism (Singh & Henrich, 2020) could show simple linear negative frequency-dependency (shamans steadily less successful as more common) or non-linear effects with mild negative effects on fitness for the first few shamans per tribe (as the niche fills up) and strong negative effects beyond that (once the niche is filled), or even positive frequency-dependency for the first few shamans (whose mutual presence reify their special status) and negative frequency-dependency beyond that. Negative frequency-dependency may affect niche occupiers differently, if the costs of increasing frequency are borne by certain individuals more than others (e.g. if the top shaman earns stable benefits despite niche filling, but lesser shamans compete for fixed benefits; Singh & Henrich, 2020). Even a particular trait's dynamics could differ between populations and over time, affecting the adaptive solutions and roles for fixed and plastic effects.

Anthropologists have not generally aimed to describe social niches and individual cognitive specialisation (perhaps excepting shamanism, e.g. Lightner et al., 2021a; Singh, 2017; Winkelman, 1990), concentrating more on age and sex differences. As modern studies consistently find that individual differences in personality and psychopathology are larger than group differences, this could be an area of important future research in traditional-living populations. Considering the non-human examples and theoretical requisites, the available psychological and anthropological evidence implies human evolutionary history would serve as fertile ground for the evolution of specialised minds, despite a lack of specific investigation into this question hitherto.

## 4. Adaptation and maladaptation in human personality

Evolutionary explanations of individual differences in psychology have largely concentrated on personality (Buss & Hawley, 2011). Mainstream personality psychology currently centres around factor-analytically derived traits representing clusters of co-occurring common personality descriptors. This method is inspired by the lexical hypothesis of personality, that socially important personality characteristics should be labelled and thereby appear in a language's vocabulary. Importantly, items included for factor analysis are chosen by psychologists, so do not include every stable psychological individual difference of evolutionary relevance. For example, variation in sociosexuality is absent, despite potentially being an adaptive individual difference under negative frequency-dependent selection or adaptive developmental plasticity (Bailey et al., 2000).

The most prominent model describing such personality factors is the five-factor ‘Big Five’ model (McCrae & Costa, 1997), identifying dimensions of openness, conscientiousness, extraversion, agreeableness and neuroticism. These traits are relatively stable over the life course (Graham et al., 2020) and affect various outcomes, including suitability to different careers (Denissen et al., 2018; Wilmot & Ones, 2019) and sexual behaviours and fertility (Allen & Robson, 2018; Allen & Walter, 2018). The Big Five has long been touted as a human universal (McCrae & Costa, 1997) and has been replicated in over 50 countries (Schmitt et al., 2007), although a more recent study in a small-scale subsistence society failed to replicate the same factor structure (Gurven et al., 2013) and large-scale surveys in 23 low- and middle-income countries have found the common Big Five Inventory lacks validity (Laajaj et al., 2019), probably because of methodological issues in translation, wording, interpretation and response biases. Sex differences in Big Five personality are generally small, although moderate differences are seen in agreeableness and neuroticism, in which females are higher (Del Giudice, 2015; Kajonius & Johnson, 2018). The sex differences have perhaps more frequently received evolutionary analysis (e.g. Kajonius & Johnson, 2018), even though group variance is smaller than individual variance, implying that adaptive explanations of individual variance are of high importance.

Personality factors seem to be caused by moderate genetic and environmental components, with an estimate of 40–50% heritability (Polderman et al., 2015; Vukasović & Bratko, 2015). Some of this variance is probably neutral or deleterious, with adaptive effects arising via both balancing selection on genes and developmental plasticity (Penke, 2010; Penke & Jokela, 2016). A prominent alternative explanation appeals to ‘reactive heritability’ (Lukaszewski & Roney, 2011) – other physical traits are heritable, such as body size, height and attractiveness, and personality could be a universal facultative programme reacting to these physical differences. Extraversion has been proposed as such a facultative strategy (Lukaszewski & von Rueden, 2015). In testing this hypothesis, Haysom et al. (2014) found that attractiveness accounted for only a small percentage of variance in extraversion, and other predicted reactive heritability effects were not found. Balancing selection thus probably partially explains personality's heritability (Buss & Hawley, 2011). Analysing the Big Five, Nettle (2006, 2011) identified possible costs and benefits causing balancing selection (Table 1). For example, high extraversion is associated with larger cooperative networks, increased mating success and positions of social status and leadership. However, it also increases the likelihood of experiencing antagonistic conflict, illnesses and injuries (Lukaszewski & von Rueden, 2015), implying variable benefits and costs in hunter-gatherer bands dependent on group composition and specific environmental circumstances.

**Table 1.** Plausible benefits and costs of the Big Five, derived from Nettle (2006, 2011)

Factor	Benefits	Costs
Openness	Artistic creativity; intellectual curiosity	Disorganised thought; tendencies to schizotypy and schizophrenia
Conscientiousness	Self-control; perfectionism; care in premeditated tasks; moral principle	Pathological levels of effort; rigidity; lack of spontaneity
Extraversion	Larger cooperative networks; high status; mating success	Increased risk of social conflict; illness and injury
Agreeableness	Empathy; helpfulness; harmonious interpersonal relationships	Vulnerable to cheaters; suboptimising of personal fitness
Neuroticism	Wariness; vigilance; useful anxiety	Debilitating anxiety; depression; physical health costs

Important in linking personality to psychopathology is the recognition that Big Five personality dimensions are associated with specific disorders and generally maladaptive outcomes, often at their tails. High neuroticism in particular is associated with anxiety, mood, eating, somatic symptom and substance use disorders, as well as social and specific phobias and various physical maladies (Widiger et al., 2018). A review of over 150 studies investigating how the Big Five relates to personality disorders (PDs) (Widiger et al., 2017) reveals the extent to which personality and psychopathology are related, with both poles of each factor associated with maladaptive outcomes. High extraversion is associated with histrionic PD, high conscientiousness with obsessive–compulsive PD and high agreeableness with dependent PD. Low extraversion is associated with schizoid PD and avoidant PD, low conscientiousness with antisocial PD and laxness, negligence, disinhibition and irresponsibility, and low agreeableness with antisocial PD and the ‘dark triad’ traits of psychopathy, narcissism and Machiavellianism. It should be noted that such correlations are instrument dependent (Widiger et al., 2017). The fifth edition of the *Diagnostic and statistical manual of mental disorders* (DSM-5) in Section III, for ‘emerging measures and models’ (American Psychiatric Association, 2013: 728), included a dimensional model of five broad domains of negative affectivity, detachment, psychoticism, antagonism and disinhibition, explicitly recognising ‘these five broad domains are maladaptive variants of the five domains of the extensively validated and replicated personality model known as the “Big Five”’ (American Psychiatric Association, 2013: 773).

Despite success linking the Big Five with certain maladaptive outcomes and particular disorders, the totality of common psychopathological traits cannot merely be reduced to aspects of the Big Five. Even recent attempts to create entirely dimensional classifications of mental disorders using factor-analytic methods from personality psychology applied to mental disorder questionnaires fail to incorporate prominent conditions such as autism spectrum disorder (Kotov et al., 2017). The 157 diagnoses in the DSM-5 may overlap and correlate with other personality measures and disorders, but certainly require their own analysis.

## 5. Evolutionary accounts of psychopathology

**Figure 1.**
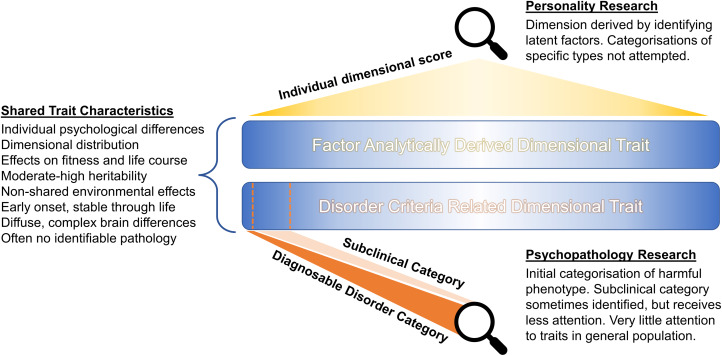
Traits of personality and psychopathology traits share basic characteristics but are studied differently.

Personality and psychopathology are described using different methodologies owing to psychiatry and clinical psychology's requirement for categorical diagnoses to inform treatment decisions (see Figure 1). Modern personality psychology is explicitly dimensional, not attempting to separately categorise particular personality types (although this is common outside of mainstream personality psychology, e.g. Myers–Briggs types). Despite the clinical necessity of discrete categorisation, psychopathologies also exist on dimensional spectra (Kotov et al., 2017; Widiger et al., 2017), with symptoms/traits visible in the general and subclinical population, especially among family members. Disciplinary separation obfuscates the many shared biological and epidemiological characteristics between personality and psychopathology dimensions (Figure 1). Brain differences are diffuse and complex (Latzman et al., 2021) and heritability is often moderate to high (Polderman et al., 2015). Environmental components seem to be mainly non-shared (Plomin, 2011). Traits are (by definition) observable relatively early and stable throughout life, with effects on fitness (Allen & Robson, 2018; Power et al., 2013), and are common, both in clinically diagnosable and subclinical trait form, which is highly significant for evolutionary explanations (see Section 7). Most individual differences in personality or psychopathology have no discernible pathological cause. Beyond socially ordained descriptive differences and the presence of apparent harm, these traits are naturalistically similar.

Psychopathologies and their evolutionary accounts are too numerous to list here (see Del Giudice, 2018 for a comprehensive review). No single evolutionary explanation is applied between disorders (Table 2), and some accounts rely on less well substantiated evolutionary mechanisms (e.g. group selection) than are focussed on in the (human or non-human) personality literature. Possible costs and benefits of the traits are often emphasised. A point made near universally in the evolution-of-personality but less regularly in the psychopathology literature is of individual differences as *themselves* adaptive. Psychopathology accounts more often hypothesise that differential susceptibility results from simple dysfunction. Key points of five accounts of psychopathological traits are provided in Table 2. These five accounts are selected to show the variability of models used and the range of benefits and costs proposed, not to represent consensus or our preference.

**Table 2.** Benefits, costs and models in a selection of evolutionary accounts for psychopathological traits

Condition	Proposed benefit	Proposed cost	Evolutionary model
Attention deficit hyperactivity disorder (ADHD) (Williams & Taylor, 2006)	Exploratory behaviour improves collective foraging, risk taking, sexual attractiveness	Physical, mental and social dangers of higher risk taking	Diversity-dependent group selection
Autism spectrum disorder (Crespi, 2016)	Species-wide positive selection on high intelligence, visuo-spatial strengths, attentional focus, deliberative decision making	Dysregulation and imbalance of intelligence causes autistic phenotypes of social, communication and behavioural difficulties	Species-wide positive selection with by-product
Bipolar disorder (Akiskal & Akiskal, 2005)	Subclinical traits beneficial (depressive sensitivity to suffering; creativity and high energy beneficial in leadership, exploration, territoriality and mating)	Extreme manifestations cause severe depression and maladaptive mania	Adaptive spectrum with costly extremes
Psychopathy (Mealey, 1995)	Antisocial strategy of emotionlessness, allowing cheating and optimally selfish behaviour, unconstrained by empathy or guilt	Possibility of detection and punishment	Negatively frequency-dependent and adaptive developmental plasticity
Schizophrenia (Polimeni & Reiss, 2002)	Religious delusions, hallucinations and social withdrawal interpreted as shamanic experiences; shamanic rituals increase group cohesion	Delirium, substance abuse, exacerbated psychotic symptoms in certain cultural contexts	Group selection for specialised roles

For two traits, autism and psychopathy, leading accounts agree on costs and benefits, but differ in details (see below). Accounts of attention deficit hyperactivity disorder (ADHD), bipolar and schizophrenia vary widely between authors. ADHD has been proposed as hunting rather than farming behaviour; or part of a ‘fighter’ strategy; or a ‘response-ready’ strategy (Del Giudice, 2018: 266–268); or adaptive at the group level by improving collective foraging (Williams & Taylor, 2006). Hypotheses of bipolar disorder associate manic states with social dominance behaviours; hypomanic states with mastery and success in technical and artistic domains; as adapted to changing climates, with depression suitable for winter and mania for spring and summer (Del Giudice, 2018: 236–238); or manic states as enhancing creativity and energy in leadership, exploration, mating and territoriality, with depressive states enhancing sensitivity to suffering (Akiskal & Akiskal, 2005). Hypotheses of schizophrenia as resulting from specialised adaptation generally concentrate on subclinical schizotypal traits or less severe cases – suggestions are of a group-splitting function to enable smooth fissioning of hunter-gatherer tribes; or as sexually selected for enhanced creativity (Del Giudice, 2018: 219–222); or as causing shamanism which enhances group cohesion (Polimeni & Reiss, 2002). By-product hypotheses of schizophrenia implicate side effects of selection for lipid metabolism which enhanced creativity, religiosity and mentalising; or failure to establish hemispheric dominance for language; or various vulnerabilities in the ‘social brain’ (Del Giudice, 2018: 217–227).

Accounts of autism spectrum disorder (ASD) show more congruence (but see Del Giudice, 2018: 251–254). ASD is diagnosed by a broad array of differences and difficulties in socialising, communication and behaviour, with significant individual variability in presentation, hence the ‘spectrum’ (Lord et al., 2020). Some cases (often associated with intellectual disability) are caused by specific genetic or environmental disruptions of normal development, requiring no evolutionary explanation beyond recognising why such vulnerabilities exist (e.g. to mutations or prenatal toxins; Del Giudice, 2018). Other cases are unexplained by such disruptions and less frequently show intellectual disability, and autistic-like traits (which can coalesce in a ‘broad autism phenotype’; Sasson et al., 2013) are more frequently seen in family members. These are the main target of evolutionary explanations. Three prominent accounts (Baron-Cohen, 2020; Crespi, 2016; Del Giudice, 2018) emphasise similar positive attributes observed in autistic and autistic-like traits. These include advantages in visual–spatial skills and abstract spatial reasoning, detail-oriented styles of cognition which boost ‘systemising’, enhanced pattern recognition, sensory acuity and perceptual discrimination and lower susceptibility to illusions (Del Giudice, 2018). Despite agreeing that ASD's evolutionary explanation revolves around these positive attributes, hypotheses differ in specifics. Baron-Cohen (2020) frames autistic traits as systemising, and autistics (without intellectual disability) as hyper-systemisers, occupying a social niche as tool-makers, inventors and experts in areas of their obsession. Sometimes this systemising overexpresses, leading to more disability. Del Giudice (2018) argues that autistic-like traits delay reproduction and increase parental investment as a slow life history strategy, whilst accumulating embodied capital through specialised skills learning, leading to a skilled/provisioning strategy. Maladaptive cases arise from overexpression of potentially adaptive traits, exacerbated by assortative mating, mutations and environmental insults. Baron-Cohen and Del Giudice therefore agree on autistic traits as specialisations, but differ on specifics of function. Crespi (2016) provides an alternative account, of ASD as a dysregulation of intelligence in components associated with strengths (e.g. visual–spatial abilities). He hypothesises that strong recent positive selection for human intelligence has led to cases of ASD as maladaptive by-products in some individuals, without claiming that the individual differences in autistic traits are themselves adaptive (although previous work (Crespi & Badcock, 2008) mentions specialised cognitive strengths and impairments in autistic and psychotic-spectrum individuals).

Psychopathy is characterised by callous and unemotional traits, impulsiveness, manipulativeness and remorselessness. Modern psychopaths can be career-focussed and manipulate their way up career hierarchies (Chiaburu et al., 2013). Aspects of psychopathy seem suitable for politics (Lilienfeld et al., 2012). Psychopathy has received substantial attention as an adaptive strategy, partly because cheating–cooperating strategies are canonical in game theory (e.g. prisoner's dilemma), inspiring early evolutionary accounts (Harpending & Sobus, 1987). Mealey (1995) suggested that psychopathy is maintained by negative frequency-dependency and adaptive developmental plasticity, with a low frequency of psychopaths (to Mealey, ‘primary sociopaths’ utilising a genetic strategy, ‘secondary sociopaths’ utilising a reactive plastic strategy) taking a ‘cheater niche’ in cooperating human groups, accurately assessing the costs and benefits of cheating to cheat as much as is personally profitable. The prevalence of full psychopathy is about 1–2% for males and 0.3–0.7% for females, with a wider psychopathic personality in about 10–12% of the population (Colins et al., 2016). Gervais et al. (2013) suggested that clinical psychopathy is a strategy of unconditional defection, whilst subclinical psychopathic traits promote strategic conditional defection, broadening the adaptive niche of psychopathy in human societies. Other authors have framed psychopathy as a fast life history strategy, noting similar benefits and costs for psychopathic traits (Barr & Quinsey, 2004; Krupp et al., 2013).

These accounts of ASD and psychopathy potentially present examples of theoretically predicted cooperating and cheating evolutionary strategies, respectively. However, in general, accounts of psychopathology are more disconnected from background theory than literature on the evolution of personality (references to group selection specifically imply this oversight), emphasising specific disorder characteristics and hypothetical costs and benefits rather than realistic evolutionary models. This may partly explain the varied accounts of schizophrenia, ADHD and bipolar disorder. We suggest that progress will come by recognising non-human and theoretical models of adaptive individual differences (Section 2), better information regarding evolutionary human social dynamics (Section 3) and clearer acknowledgement of the similarity and relationship between personality and psychopathology trait dimensions (Figure 1), implying a need for shared scientific explanations despite disciplinary separation.

Previous authors have mistakenly inferred that identifiable instances of pathological processes affecting psychopathology (Keller & Miller, 2006) and personality (Verweij et al., 2012) imply that the *entirety* of variance is pathological. The fact that traumatic brain injury can cause aggressive personalities does not imply all aggressive personality is pathological – we cannot infer causation of heterogenous trait categories from causation of single instances. Predictions that mutation-selection balance would explain all heritable variance in disorders have not borne out (Keller, 2018; Keller & Miller, 2006). Pure by-product or pathology explanations of heterogenous phenotype dimensions such as ASD are bound to fail to explain the full clinical and subclinical variance. Similarly, adaptive specialisation clearly will not explain cases associated with *de novo* mutations. Every psychopathology dimension probably contains pathological variance (probably the most debilitating cases) and adapted variance (probably subclinical cases). Better recognition of this heterogeneity and of the plausible evolutionary models of personality adaptation offers promise for guiding evolutionary explanations of psychopathological traits.

## 6. Minimum adaptive prevalence

To offer some novel contribution beyond the review and commentary above, we now briefly sketch an example of practical synthesis across these areas. This specifically talks to a longstanding problem in the philosophy of medicine, involving the validity of attributing disorder to statistical outliers (Rogers & Walker, 2017). Evolution- and anthropology-informed work could certify low-frequency traits as disorders – or at least, not adaptations – with naturalistic rather than arbitrary justification of line-drawing at a particular prevalence.

We introduce this as a conceptual prompt for future research rather than an immediately applicable model. We encourage formal modelling and recognise that specific empirical details of the trait being analysed will need accounting for, including onset and duration, dimensionality, genetic architecture, environmental effects and probably selection dynamics (as noted in various plausible shamanism niche dynamics in Section 3). Nevertheless, we believe that framing this fundamental concept offers hope of progress in assessing psychopathology evolutionarily, and that further work can even be extrapolated to understanding specialisation more generally, especially in illuminating function, rather than simply providing a lower bound frequency for specialised adaptations.

Negative frequency-dependency is a key force of balancing selection, regularly included in models of stable individual differences, including niche specialisation. When negatively frequency-dependent phenotypes reduce in frequency, negative selection effects approach 0. Upon reaching 1 individual with a phenotype, negative selection reaches 0 (to apply to dimensional models assume that different positions on the dimension are different phenotypes). Negative frequency-dependence can thus maintain, at an absolute minimum, one individual with a specialised phenotype (or capacity to develop a phenotype) per relevant group of interacting conspecifics. This allows dimensional tails to be deemed almost certain non-adaptations at a non-arbitrary point – when a phenotype is too rare to exist in every interacting group. So, where negative frequency-dependency is maintaining specialisation, we can derive a minimum adaptive prevalence (MAP) for a specialised trait using evolutionarily relevant group sizes. Where group size is represented as *G*, the simplest estimate of MAP is calculated with the equation:

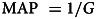

Thus, under simplifying assumptions, in species interacting in groups of 30–40, the MAP of a negatively frequency-dependent phenotype is 1/35, approximately 3%. If 1% of individuals in that species display a phenotype, we can infer that it is not a specialised trait maintained by negative frequency-dependent selection. Note the assumption of equal environmental effects; if the specialised strategy is as an adaptive plastic developmental response (e.g. only becoming a psychopath if raised in an abusive environment) or also under temporally fluctuating selection, then the observed prevalence can be lower than the MAP. Also note the importance of accounting for trait-specific features and possible functions – if the trait is sexually dimorphic (perhaps sexually selected) or shows a delayed age of onset, effective group size is reduced, because negative frequency-dependency occurs via phenotype interaction and competition – the evolutionarily relevant group is then smaller than the whole group size. Beyond ‘once per group’ specialisations, ‘once per males/females in group’ or ‘once per age group’ specialisations are plausible, but need the group size to be adjusted to the relevant population to calculate their MAP (Table 3). Phenotype competition often occurs within demographics – rarely would a human 5 year old's phenotype have negative frequency-dependent effects on a 25 year old's similar phenotype – so this is an important consideration in discerning (and excluding) potential specialisations.

**Table 3.** Estimated individuals per varying group, and resulting frequency of a particular trait given its prevalence. Zeros indicate prevalence falling below the MAP, indicating that the trait cannot be the result of a negatively frequency-dependent specialisation for that group

		Prevalence; percentage/individuals per group						
		50%	20%	10%	5%	1%	0.5%	0.2%
Group type (size) (Hamilton et al., 2007; Hill et al., 2011)	Aggregated bands (165)	82–83	33	16–17	8	1–2	0–1	0
	Males/females per aggregated band (83)	42	17	8	4	0–1	0	0
	Adult male/females per aggregated band (42)	21	8	4	2	0–1	0	0
	Band (28)	14	5–6	2–3	1	0	0	0
	Males/females per band (8–12)	4–6	2	1	0	0	0	0
	Adult male/females per band (4–6)	2–3	1	0–1	0	0	0	0

## 7. Specific prevalence of human specialised minds

Calculating the MAP for human traits requires knowledge of human social group size and dynamics. Band size is around 28 individuals, a relevant figure in assessing specialisation function, but does not provide the MAP, because bands are interconnected. Negative frequency-dependency relies upon interacting phenotypes reducing each other's fitness. The relevant social group size in estimating human MAP must interact frequently enough that individual differences interrupt each other and compete, and be sensitive enough to phenotype frequency change that one individual per group could display an adaptive phenotype, but in two individuals negative selection would reduce the phenotype's frequency back down to one individual per group. This group size will be much lower than lifetime interaction partners (which can be several hundreds; Hill et al., 2014), but could be larger than band size. Anthropologists have not specifically tackled this question, but as a placeholder for purposes of example, a reasonable estimate can be derived from periodic aggregations of bands totalling around 165 individuals (Hamilton et al., 2007). Periodic aggregations interact frequently enough, and phenotypes which do not periodically aggregate seem unlikely to negatively impact each other via their frequency. Although larger periodic aggregations have been recorded, the chance of relevant interaction in such large groups drops, and the relevant group size is that which caused selection over many generations, so the aggregate mean seems more relevant than higher or lower bounds of band aggregations (although this depends on specific selection effects and deserves separate extended inquiry). Taking shamanism as prototypical role specialisation filling a human social niche, feasibly one shaman per 165 individuals experiences optimum benefits, additional shamans experiencing negatively frequency-dependent declining benefits as the niche fills, and groups much larger than 165 probably cannot have their demand for shamanistic services met by one shaman (Singh, 2017; Singh & Henrich, 2020).

**Figure 2.**
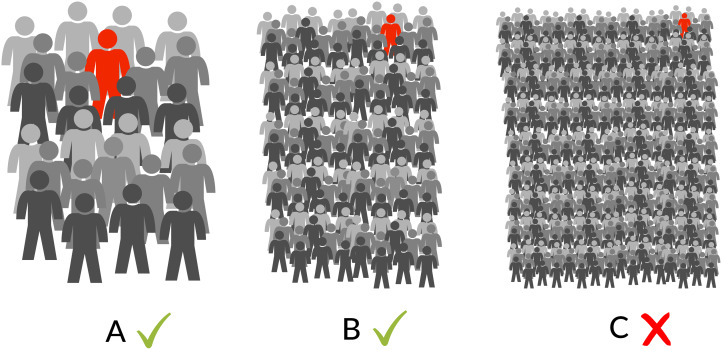
Prevalence per group size in relation to MAP. (A) is approximately band sized, 26 individuals. (B) is approximately band-aggregation sized, 130 individuals. (C) is more than twice band-aggregation size, 416 individuals. Phenotypes appearing once in (A) and (B) could plausibly be adaptive and maintained by negative frequency-dependency; phenotypes appearing once in (C) could not.

The MAP of a group of 165 is approximately 0.6% so (in this simplified conceptual model) traits rarer than 0.6% prevalence are highly unlikely to be adaptations (Figure 2). *Note this is an absolute minimum*, and traits at 0.6% prevalence are only eligible for a very specific adaptive explanation – a specialisation functional for only one individual per group of aggregating bands – presumably an unusual occurrence. Sex- or age-group specific specialisations (e.g. ‘once per adolescent males in aggregating bands’) have lower group sizes and thus a higher MAP. Because of regularity of interaction, it is perhaps more plausible that ‘one per group’ specialisations were maintained by negative frequency-dependency in adults within bands (approximately 8–12 individuals), yet exist in exaggerated maladaptive forms in every aggregation of bands (possible if the inclusive fitness cost/benefit is stable). If so, maladaptive traits related to adaptations would exist above the MAP derived from band agglomeration size – this should encourage caution against using the MAP as a simple cut-off prevalence above which adaptation is expected and below which dysfunction is evident.

**Figure 3.**
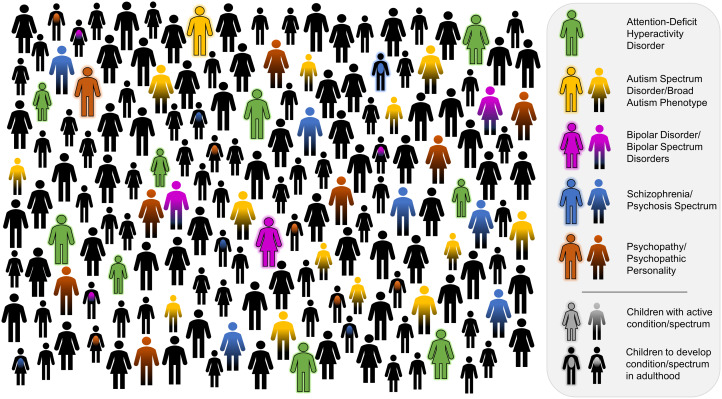
The expected prevalence of five psychopathological traits (Table 2) and their subclinical spectra amongst a band-aggregation sized population of 165 adults and children. Following diagnostic norms and clinical appearance, schizophrenia, bipolar and psychopathy are displayed as adult onset (assumed around 18 years of age); ADHD and autism are active in children. Some accounting for gender differences and comorbidity between ADHD/autism spectra (Antshel et al., 2014) and bipolar/psychosis spectra (Kotov et al., 2020) has been made. Psychopathy and psychopathic personality, Colins et al. (2016); schizophrenia, McGrath et al. (2008) and psychosis spectrum, Guloksuz & Van Os (2018); bipolar disorder and bipolar spectrum disorders, Merikangas et al. (2011); autism spectrum disorder, Lord et al. (2020) and broad autism phenotype, Sasson et al. (2013); and attention-deficit hyperactivity disorder, Polanczyk et al. (2014).

This analysis speaks to the paradox of common, harmful, heritable mental disorders (Keller & Miller, 2006), placing the ‘common’ factor in the appropriate context of ancestral human group sizes. Most common psychopathological traits (including all those mentioned in Section 5) have a lifetime prevalence of around or over 1%. Thus, every human ancestor probably interacted with at least one individual with each condition (assuming fairly constant cross-cultural rates, which is plausible, although difficult to test; Steel et al., 2014). Certain conditions and subclinical manifestations such as ADHD and broad autism phenotype have a prevalence of approximately 7% (Figure 3) – so predictably present once or twice in every band. Crucial, though, in identifying non-adaptive traits is recognising heterogeneity within psychopathology categories and specific trait characteristics. For example, some estimates of ASD base prevalence are 0.97% with approximately 40% having intellectual disability (Fombonne et al., 2021), in which case intellectually disabled cases do not meet the MAP. Lifetime schizophrenia prevalence is approximately 0.7% (although rates differ substantially between populations; McGrath et al., 2008), but schizophrenia onset is in late adolescence. Out of 165 individuals, approximately one develops schizophrenia in their life, but out of 165 hunter-gatherers, roughly half are children – one schizophrenic per two band agglomerations – so this falls below the MAP (this presents a further problem for Polimeni and Reiss's (2002) hypothesis of schizophrenia as a group-selected adaption causing shamanism). Heterogeneous genetic etiologies also require recognition. A significant proportion of cases of autism and schizophrenia are caused by rare or *de novo* variants (De La Torre-Ubieta et al., 2016; Legge et al., 2021; Singh et al., 2022), excluding adaptive explanations. Epidemiological estimates of disorder prevalence can contain potentially adaptive and certifiably dysfunctional genetic subtypes – removing certifiably dysfunctional types identifies the prevalence needed to meet the MAP. Combining these winnowing factors, the 1% lifetime prevalence of severe disorders can quickly drop below the MAP. This is compatible with hypotheses of adaptive specialisation leading to personality and psychopathology spectra, with costly extremes diagnosable as disorders.

## 8. Limitations and future directions

It is, in our view, essentially untenable that specialised minds would be predicted by theory, visible in non-humans, perfectly suited to human social dynamics and evolution, and yet not cause *any* of the variance in personality or psychopathology. Future questioning should not be whether human minds specialised at all, but uncovering the precise details and extent of that specialisation. The clearest areas for development are in fundamental theory – developing formal models accounting for the complexity of specialisation – and better empirical understanding of relevant human psychology and ancestral social dynamics – particularly in anthropology.

Calls for adaptationist explanations of psychological individual differences are plentiful (Buss & Hawley, 2011), but heterogenous and complex causation makes specific hypotheses hard to prove or assess using a rigorous scientific method (although see Hunt, in preparation). We suggest that a first crucial step in untangling heterogeneity, especially in assessing psychopathology, will follow approaches such as Del Giudice's (2018), recognising subtypes eligible for functional and dysfunctional explanations before attempting to assess adaptation. This will probably broadly redraw the line between psychiatric and neurological disorders (Anttila et al., 2018).

Specialisation should be selected for specific social niches and group dynamics – probably dramatically changed since relevant periods of human evolution, and thus less amenable to the usual methods in evolutionary psychology seeking to describe and test complex design (Lukaszewski et al., 2020; Tooby & Cosmides, 1996). Human universals governing sexual attraction, status or anger may be straightforwardly experimentally testable in developed societies, but adaptive functions of individual differences in personality may manifest unusually owing to environmental factors, and certain culturally specific group dynamics (e.g. surrounding shamanism) have probably completely changed. Any fitness effects measured in developed societies are apt to be misleading, as evidenced by dramatic drops in fertility observed in recent decades; only if fertility has dropped equally irrespective of personality and psychopathology since relevant evolutionary time periods would such measures be useful. The gap between ancestral and modern environments also forces close consideration of heritability estimates of these traits, as measured heritability depends on socio-ecological circumstances (Uchiyama et al., 2021) and indirect genetic effects from social partners (Martin & Jaeggi, 2021). Since gene–environment interactions are important in personality and psychopathology phenotypic expression, their current manifestation, population variation and heritability may differ substantially from relevant periods of evolutionary history.

For these reasons, particular importance lies in studying individual psychological differences in hunter-gatherer and other non-industrialised societies, ideally measuring fitness consequences, but at minimum assessing how (and whether) such traits manifest and function in these groups. The fact that anthropologists have concentrated on measuring and analysing overall trends, such as sex or age differences – negligible in comparison with individual differences in modern society – implies that a wealth of relevant information awaits discovery. Inter-individual differences may be pivotal to the division of labour and hunter-gatherer cooperative dynamics. An example of low-hanging fruit might be re-analyses of research on hunter-gatherer behaviour, examining variance. Models of human individual differences in behaviour as noise awaiting negative selection predict trait clustering around the optimum (probably the mean) and negative effects moving away from it. Specialisation predicts a spectrum of behavioural variation related to fixed and plastic optimisation within niches to achieve optimum inclusive fitness – useful differences, not simply noise. The fact that personality and psychopathology play such critical roles in modern life paths may not be an artefact of modernity, but a cross-culturally verifiable result of evolutionary history.
